# Protein phosphatase 4 (PP4) functions as a critical regulator in tumor necrosis factor (TNF)-α-induced hepatic insulin resistance

**DOI:** 10.1038/srep18093

**Published:** 2015-12-15

**Authors:** Hongye Zhao, Xiuqing Huang, Juan Jiao, Hangxiang Zhang, Jin Liu, Weiwei Qin, Xiangyu Meng, Tao Shen, Yajun Lin, Jiaojiao Chu, Jian Li

**Affiliations:** 1 Graduate School of Peking Union Medical College and Chinese Academy of Medical Sciences, Beijing 100730, China; 2 The Key Laboratory of Geriatrics, Beijing Hospital & Beijing Institute of Geriatrics, Ministry of Health, Beijing 100730, China; 3 College of Life Sciences, Beijing Normal University, 100875, P.R. China

## Abstract

Protein phosphatase 4 (PP4) was shown to participate in multiple cellular processes, including DNA damage response, cell cycle and embryo development. Recent studies demonstrated a looming role of PP4 in glucose metabolism. However, whether PP4 is involved in hepatic insulin resistance remains poorly understood. The objective of this study was to estimate the role of PP4 in tumor necrosis factor (TNF)-α-induced hepatic insulin resistance. db/db mice and TNF-α-treated C57BL/6J mice were used as hepatic insulin resistance animal models. *In vitro* models were established in both HepG2 cells and primary hepatocytes by TNF-α treatment. We found that increased expression and activity of PP4 occurred in the livers of db/db mice and TNF-α-induced hepatic insulin resistance both *in vitro* and *in vivo*. Actually, PP4 silencing and suppression of PP4 activity ameliorated TNF-α-induced hepatic insulin resistance, whereas over-expression of PP4 caused insulin resistance. We then further investigated the prodiabetic mechanism of PP4 in TNF-α-induced insulin resistance. We found that PP4 formed a complex with IRS-1 to promote phosphorylation of IRS-1 on serine 307 via JNK activation and reduce the expression of IRS-1. Thus, PP4 is an important regulator in inflammatory related insulin resistance.

Insulin resistance, defined as a diminished ability of cells, such as adipocytes, skeletal muscle cells and hepatocytes, to respond to the action of insulin, plays a central role in the development of obesity, type 2 diabetes and the metabolic syndrome^1^. Decreased glycogen level is a hallmark of insulin resistance in the hepatocytes and the underlying mechanisms include decreased glycogen synthesis and failure to suppress glucose production^2^.

Chronic inflammation plays a key role in the pathogenesis of insulin resistance. The cytokines such as tumor necrosis factor (TNF)-α promotes inflammation and suppresses insulin sensitivity in insulin target cells^3^,^4^. TNF-α has been implicated in the pathogenesis of insulin resistance *in vitro* and *in vivo*. Elevated plasma TNF-α levels may be an important mediator of insulin resistance by impairing insulin signaling^5^. Our previous study also indicated that in HepG2 hepatocytes, TNF-α induced insulin resistance, as assessed by their decreased capacity to accumulate glycogen in the presence of insulin^6^. However, the mechanisms linking TNF-α to hepatic insulin resistance remain poorly understood.

Protein phosphatase 4 (PP4), a PP2A-like phosphatase, was shown to participate in multiple cellular processes as diverse as organelle assembly, DNA damage response, cell cycle and embryo development^7^,^8^,^9^. Also PP4 could regulate multiple signal transductions, especially TNF-α initiated pathway^10^,^11^,^12^. Zhou et.al identified that PP4 mediated TNF-α-induced activation of JNK^10^. Later, PP4 was found to participate in HPK signaling which is one of the upstream regulators of JNK^12^. More important, it was reported a role of PP4 in TNF-α-induced down-regulated expression of IRS-4^11^. Moreover, recent studies demonstrated a looming role of PP4 in glucose metabolism. SMEK/PP4C proteins were considered to involve in the regulation of hepatic gluconeogenesis to maintain glucose homeostasis^13^. In yeast, Pph3-Psy2 (mammalian PP4C-R3 complex) was specifically involved in glucose signaling-mediated HXT (induction of glucose transporter) gene expression, suggesting that PP4 might participate in glucose metabolism^14^. However, whether PP4 is involved in TNF-α-induced hepatic insulin resistance remains unclear.

In the current study, we examined the role of PP4 in TNF-α-induced hepatic insulin resistance both *in vivo* and *in vitro*. Moreover, we explored the mechanism by which PP4 involve in the insulin signaling pathway.

## Results

### Increased expression and activity of PP4 occurred in the livers of db/db mice and TNF-α-induced hepatic insulin resistance both *in vitro* and *in vivo*

**Figure 1:**
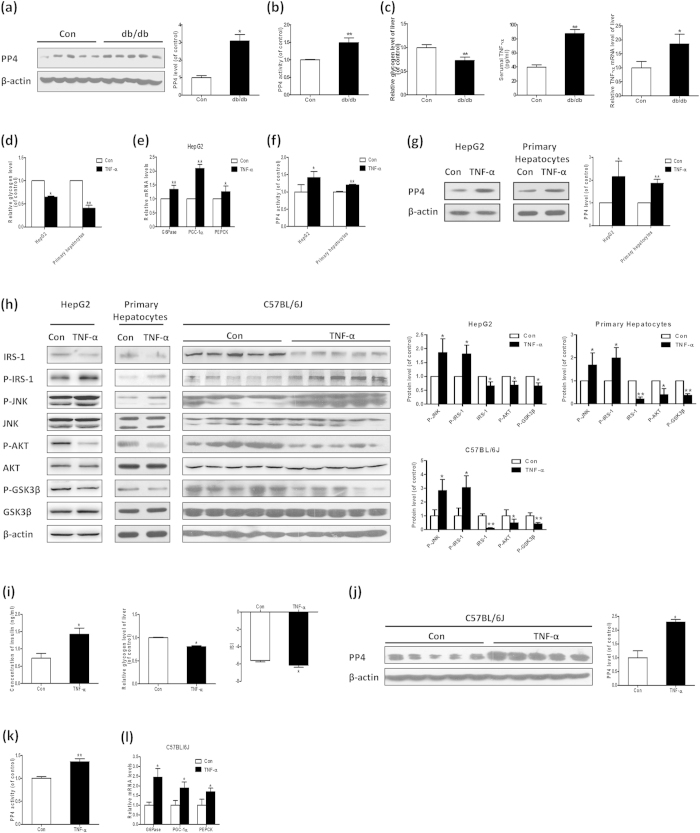
Increased expression and activity of PP4 occurred in the livers of db/db mice and TNF-α-induced hepatic insulin resistance both *in vitro* and *in vivo*. The expression (**a**) and activity (**b**) of PP4 is increased, accompanied by decreased content of hepatic glycogen and increased serumal and hepatic level of TNF-α (**c**) in db/db mice. In HepG2 cells and mouse primary hepatocytes treated with TNF-α, the insulin signaling pathway is disordered (**h**), acompanied with decreased glycogen content (**d**) and elevated mRNA levels of glyconeogenesis-related genes (**e**). The activity (**f**) and expression (**g**) of PP4 is also elevated. Similar results were obtained in C57BL/6J mice treated with TNF-α (**h**,**i–l**). Data represent the means ± S.D., (n = 3 independent experiments *in vitro*; n = 10 mice *in vivo*). *P < 0.05 and **P < 0.01 versus control. Con, control.

The type 2 diabetic db/db mice exhibited typical symptoms of diabetes, including increased levels of blood glucose, hyperinsulinemia, decreased insulin sensitivity index (see Supplementary Fig. S1a–c). Moreover, the insulin signaling pathway was disordered in the livers (see Supplementary Fig. S1d), characterized by increased IRS-1 (serine 307) and JNK phosphorylation, reduced IRS-1 expression and AKT/GSK3β phosphorylation. Also, db/db mice showed decreased content of hepatic glycogen (Fig. 1c). Interestingly, the expression and activity of PP4 is increased in the livers (Fig. 1a,b), suggesting that PP4 might be involved in the pathogenesis of hepatic insulin resistance in db/db mice. These observations were extended to more specific models of hepatic insulin resistance. Based on TNF-α is recognized as a key mediator in hepatic insulin resistance and increased level of TNF-α was detected in the serum and livers of db/db mice (Fig. 1c), HepG2 cells and cultured primary hepatocytes were treated with 10 ng/ml TNF-α for 24 h, which exhibited obvious hepatic insulin resistance, as assessed by decreased intracellular glycogen content (Fig. 1d), elevated mRNA levels of glyconeogenesis-related genes, such as phosphoenolpyruvate carboxykinase (PEPCK), glucose-6-phosphatase (G6Pase) and peroxisome proliferator-activated receptor gamma coactivator 1alpha (PGC-1α) in HepG2 cells (Fig. 1e) and disordered insulin signaling pathway (Fig. 1h). Moreover, the activity and expression of PP4 is elevated in the hepatocytes treated with TNF-α (Fig. 1f,g). Similar results were obtained in C57BL/6J mice treated with TNF-α (Fig. 1h,i–1). These data demonstrated that TNF-α could induce hepatic insulin resistance and up-regulate the expression and activity of PP4 both *in vitro* and *in vivo*.

### Knock-down of PP4 restores TNF-α-induced hepatic insulin resistance *in vitro* and *in vivo*

**Figure 2:**
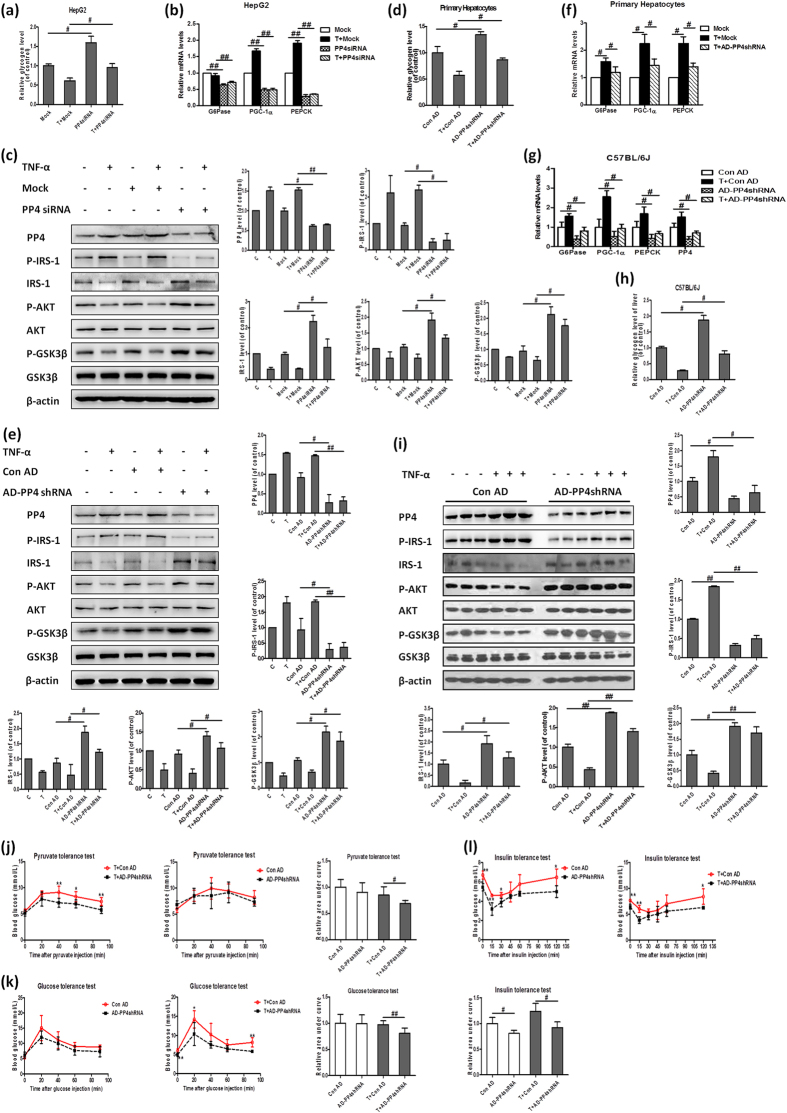
Knock-down of PP4 restores TNF-α-induced hepatic insulin resistance *in vitro* and *in vivo*. Knock-down of PP4 led to increased glycogen content in HepG2 cells (**a**), decreased mRNA levels of glyconeogenesis-related genes (**b**) and disordered insulin signaling pathway (**c**). These results were further confirmed in cultured mouse primary hepatocytes by AD-PP4 shRNA transfection (**d–f**). AD-PP4 shRNA was injected into the tail veils of C57BL/6J mice treated with or without TNF-α. PP4 suppression could restore impaired insulin signaling (**i**), reduce mRNA levels of PP4 and glyconeogenesis-related genes (**g**) as well as increase glycogen level of liver (**h**). Pyruvate tolerance tests (**j**), glucose tolerance tests (**k**) and insulin tolerance tests (**l**) were performed as described in Methods. Data represent means ± S.D. (n = 3 independent experiments *in vitro*; n = 9 mice *in vivo*). *P < 0.05 and **P < 0.01 versus control; ^**#**^P < 0.05 and ^**##**^P < 0.01; the 2 groups are as indicated by the line. C, control; T, TNF-α; Con AD, control AD.

To assess the role of PP4 in TNF-α-induced hepatic insulin resistance, PP4 siRNA was transfected into HepG2 cells (see Supplementary Fig. S2a,b). Silencing of PP4 led to increased glycogen content, especially in the HepG2 cells treated with TNF-α (Fig. 2a). The insulin signaling pathway was ameliorated, as evidenced by reduced IRS-1 (serine 307) phosphorylation, elevated IRS-1 expression and AKT/GSK3β phosphorylation (Fig. 2c). PP4 knockdown in HepG2 cells also recused TNF-α-induced increased mRNA levels of glyconeogenesis-related genes including PEPCK, G6Pase and PGC-1α (Fig. 2b). These results were further confirmed in mouse primary hepatocytes (Fig. 2d–f and Supplementary Fig. S2c,d). To validate whether PP4 silencing could ameliorate TNF-α-induced hepatic insulin resistance *in vivo*, we injected AD-PP4 shRNA into the tail veils of C57BL/6J mice treated with or without TNF-α. Suppression of PP4 could restore TNF-α-induced impaired insulin signaling, increase hepatic glycogen content as well as reduce mRNA levels of glyconeogenesis-related genes (Fig. 2g–i). Importantly, pyruvate tolerance was ameliorated in the AD-PP4 shRNA-injected mice (Fig. 2j), indicating ameliorated gluconeogenesis. Similarly, glucose tolerance tests and insulin tolerance tests indicated ameliorated glucose tolerance and improved insulin sensitivity upon PP4 suppression in the C57BL/6J mice treated with TNF-α (Fig. 2k,l). Taken together, these data suggested that knock-down of PP4 could restore TNF-α-induced hepatic insulin resistance.

### Inhibition of PP4 activity ameliorates TNF-α-induced insulin resistance

**Figure 3:**
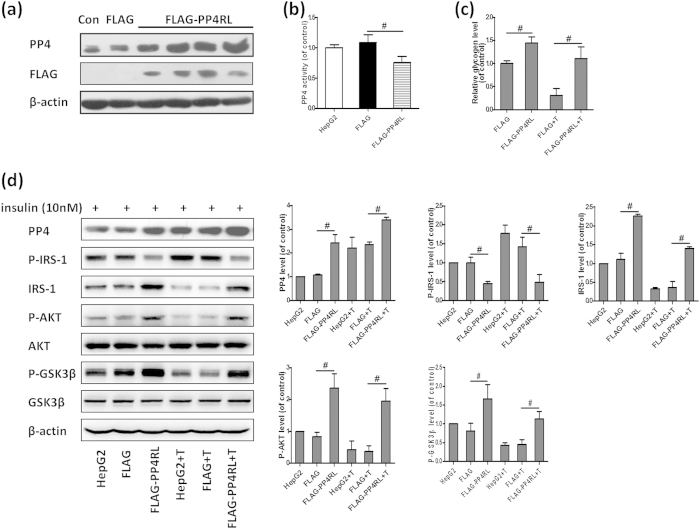
Inhibition of PP4 activity ameliorates TNF-α-induced insulin resistance. By transfecting a FLAG-tagged PP4RL expression vector into HepG2 cells, we obtained several clonal cell lines (**a**) and PP4 activity was inhibited (**b**). Inhibition of PP4 activity ameliorated reduced glycogen levels induced by TNF-α in HepG2 cells (**c**) and reversed changes in the insulin signaling pathway induced by TNF-α (**d**). Data represent means ± S.D. (n = 3 independent experiments). ^#^P < 0.05; the 2 groups are as indicated by the line. Con, control; T, TNF-α.

We next further investigated the role of PP4 activity in TNF-α-induced hepatic insulin resistance. By transfecting a FLAG-tagged PP4-RL (a PP4 phosphatase-dead mutant) expression vector into HepG2 cells, we obtained several clonal cell lines in which PP4RL was stably over-expressed and the activity of PP4 was inhibited (Fig. 3a,b). PP4RL over-expression ameliorated reduced glycogen levels induced by TNF-α in HepG2 cells (Fig. 3c). Moreover, inhibition of PP4 activity reversed changes in the insulin signaling pathway induced by TNF-α (Fig. 3d). Our data strongly suggested that PP4 might play a distinct role in TNF-α-induced hepatic insulin resistance.

### Over-expression of PP4 induces insulin resistance in hepatocytes

**Figure 4:**
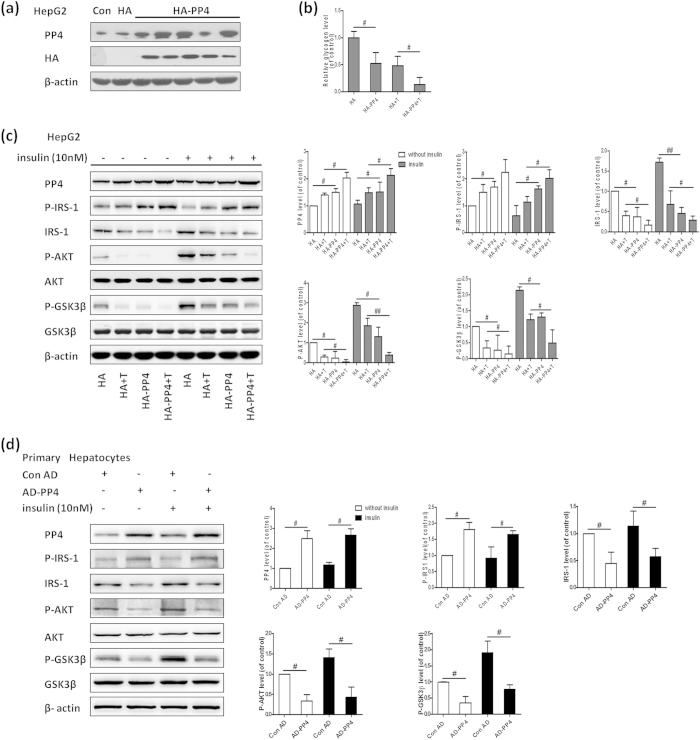
Over-expression of PP4 induces insulin resistance in hepatocytes. By transfecting the PCMV5-HA-PP4 expression vector into HepG2 cells, we obtained several clonal cell lines (**a**). PP4 over-expression resulted in reduced glycogen levels (**b**) and impaired activation of the AKT/GSK3β pathway (**c**). Similar results were obtained from AD-mediated PP4 over-expression in mouse primary hepatocytes (**d**). Data represent means ± S.D. (n = 3 independent experiments). ^#^P < 0.05 and ^**##**^P < 0.01; the 2 groups are as indicated by the line. T, TNF-α; Con, control; Con AD, control AD.

We further observed the effect of PP4 over-expression on hepatic insulin resistance. By transfecting the PCMV5-HA-PP4 expression vector into HepG2 cells, we obtained several clonal cell lines in which PP4 was stably over-expressed (Fig. 4a). PP4 over-expression resulted in reduced glycogen levels in both unstimulated and stimulated HepG2 cells (Fig. 4b). As expected, PP4 over-expression impaired the insulin signaling pathway in the HepG2 cells with or without insulin stimulation (Fig. 4c). Similar results were obtained from AD-mediated PP4 over-expression in mouse primary hepatocytes (Fig. 4d).

### PP4 regulates IRS-1 phosphorylation via JNK activation

**Figure 5:**
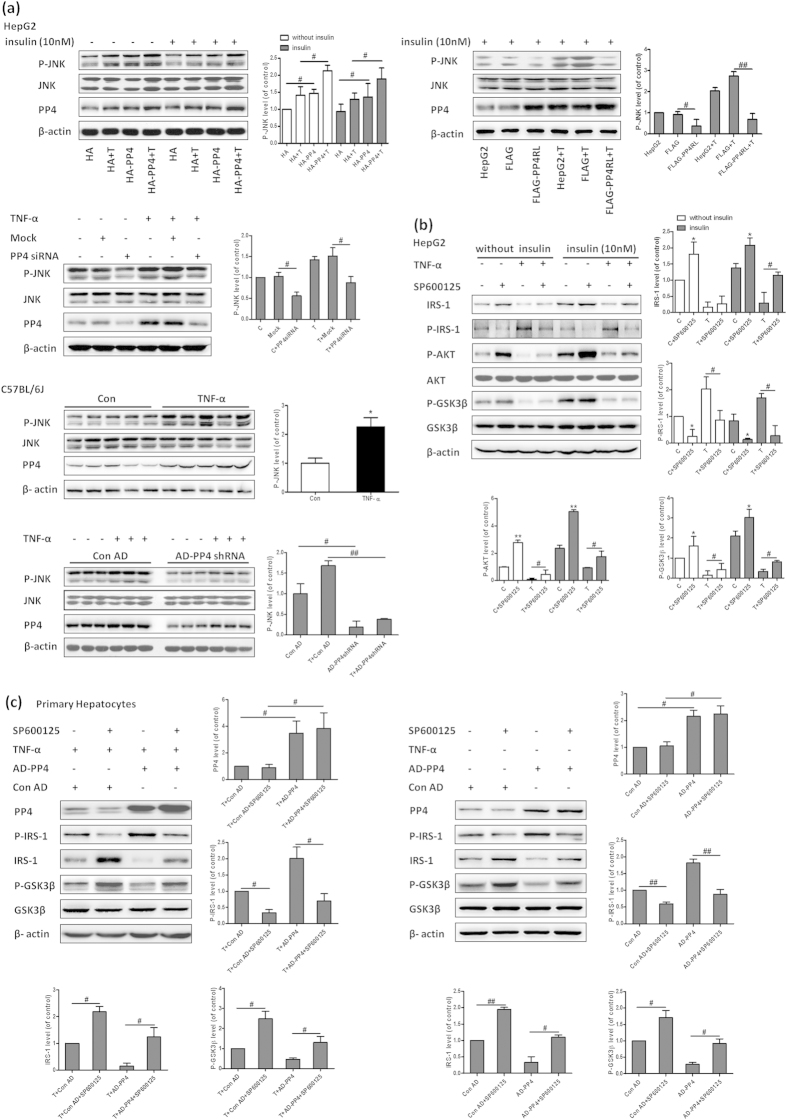
PP4 regulates IRS-1 phosphorylation via JNK activation. In HepG2 cells and the livers of C57BL/6J mice, over-expression of PP4 resulted in elevated phosphorylation level of JNK, whereas down-regulation of PP4 and over-expression of PP4RL restored increased phosphorylation of JNK induced by TNF-α (**a**). SP600125, a JNK signaling inhibitor, restored the aberrant insulin signaling induced by TNF-α (**b**). The IRS-1 serine 307 phosphorylation induced by PP4 over-expression was strongly inhibited by SP600125 (**c**). Data represent means ± S.D. (n = 3 independent experiments *in vitro*; n = 6 mice *in vivo*). *P < 0.05 and **P < 0.01 versus control; ^**#**^P < 0.05 and ^**##**^P < 0.01; the 2 groups are as indicated by the line. C, control; T, TNF-α; Con AD, control AD.

Serine 307 phosphorylation of IRS-1 has been suggested to be an important negative regulator of hepatic insulin signaling. We further assessed how PP4 regulated the phosphorylation of IRS-1. Our results indicated that the phosphorylation level of JNK was regulated by PP4. In HepG2 cells and the livers of C57BL/6J mice, over-expression of PP4 resulted in elevated phosphorylation level of JNK, whereas down-regulation of PP4 and over-expression of PP4RL restored increased phosphorylation level of JNK induced by TNF-α (Fig. 5a). Furthermore, we investigated the role of JNK in IRS-1 phosphorylation and subsequent pathway activation. SP600125, a JNK signaling inhibitor, restored the aberrant insulin signaling induced by TNF-α (Fig. 5b). Importantly, the IRS-1 phosphorylation induced by PP4 over-expression was strongly inhibited by SP600125 (Fig. 5c), suggesting that JNK mediated PP4-induced serine phosphorylation of IRS-1. However, no direct interaction was detected between PP4 and JNK (data not shown), suggesting that PP4 might regulate JNK in an indirect manner. Taken together, it is likely that PP4 regulated IRS-1 phosphorylation via JNK activation.

### PP4 forms a complex with IRS-1 to involve in the insulin signaling pathway

**Figure 6:**
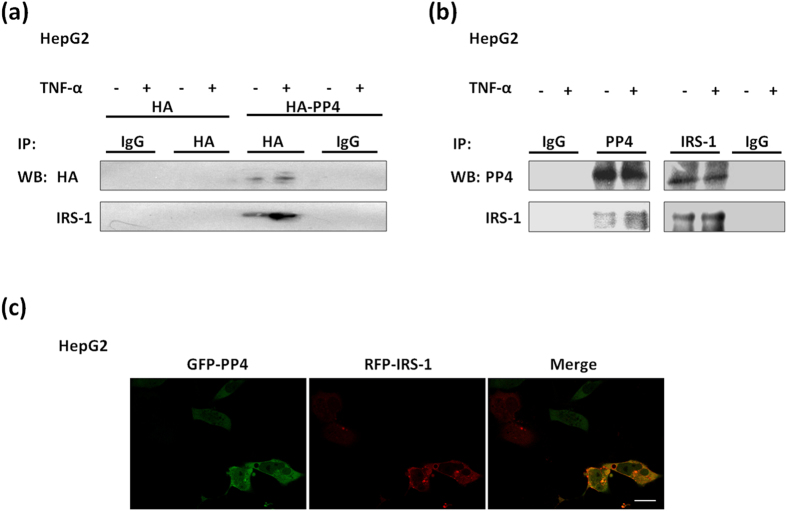
PP4 forms a complex with IRS-1 to involve in the insulin signaling pathway. When HA-tagged PP4 was transfected into HepG2 cells, endogenous IRS-1 was coimmunoprecipitated using the HA antibody (**a**). Endogenous IRS-1 was coimmunoprecipitated with endogenous PP4 in HepG2 cells (**b**). PP4 co-localized with IRS-1 in HepG2 cells (**c**). Bar = 20 μm.

Report showed that PP4C could interact with IRS-4 and down-regulate IRS-4^11^, we wondered whether PP4 was involved in TNF-α-induced insulin resistance by interacting with IRS-1. When HA-tagged PP4 was transfected into HepG2 cells, endogenous IRS-1 was coimmunoprecipitated using the HA antibody (Fig. 6a). To exclude potential artificial interactions due to the over-expression system, we investigated whether endogenous PP4 interacted with endogenous IRS-1. As shown in Fig. 6b, endogenous IRS-1 was coimmunoprecipitated with endogenous PP4 in HepG2 cells. Furthermore, we found that PP4 co-localized with IRS-1 in HepG2 cells (Fig. 6c). These observations showed that PP4 might form a complex with IRS-1 to involve in the insulin signaling pathway.

## Discussion

In the present study, we identified PP4 as a novel regulator in hepatic insulin resistance induced by TNF-α. Our study demonstrated increased expression and activity of PP4 in the livers of db/db mice, and HepG2 cells, primary hepatocytes and the livers of C57BL/6J mice treated with TNF-α. Hepatic knock-down of PP4 *in vitro* and *in vivo* as well as suppression of PP4 activity ameliorated TNF-α-induced insulin resistance, whereas over-expression of PP4 promoted insulin resistance. Moreover, we provided evidence for a possible mechanism underlying the regulation of the insulin signaling by PP4. We found that PP4 impaired the insulin signaling by forming a protein complex with IRS-1 to promote IRS-1 phosphorylation on serine 307 via JNK activation and reduce expression of IRS-1. To our knowledge, this is the first demonstration that PP4 functions as a linker between chronic inflammation and hepatic insulin resistance.

Insulin resistance may occur in major metabolic tissues, including muscle, adipose tissue and liver. More importantly, the liver is an essential organ for glucose homeostasis. Insulin signaling plays an important role in this dual nature by promoting the activation of glycogen synthesis-related genes and inhibiting gluconeogenesis-related genes. Insulin resistance is induced by several molecules, including glucose, insulin, free fatty acids (FFAs) and certain cytokines, such as TNF-α^15^,^16^. Accumulating evidence suggested that TNF-α impaired hepatic insulin sensitivity and glucose regulation both *in vitro* and *in vivo*^17^,^18^.

In the present study, we observed increased levels of TNF-α both in serum and liver, decreased hepatic glycogen content in db/db mice. The insulin signaling pathway was also impeded in the livers with obviously elevated expression and activity of PP4. These observations raise the possibility that PP4 might participate in the initiation and development of hepatic insulin resistance. Because db/db mice are complex and may exhibit alterations not restricted to the livers, it is difficult to determine the contribution of PP4 to hepatic insulin resistance. Therefore, the findings in db/db mice need to be assessed *in vitro* and *in vivo*. Consistent with previous studies^5^,^19^, treatment with TNF-α resulted in reduced glycogen content and impaired insulin signaling both in HepG2 cells and mouse primary hepatocytes. Similar data were obtained from the C57BL/6J mice treated with TNF-α. Notably, our results revealed that the expression and activity of PP4 were up-regulated in response to TNF-α stimulation. These observations indicated that PP4 might involve in TNF-α-induced hepatic insulin resistance.

It has been proposed that PP4 plays important roles in a variety of cellular processes, such as microtubule growth/organization, apoptosis, DNA replication and multiple signaling pathways^7^,^8^,^9^,^20^,^21^,^22^. Studies revealed that PP4 might participate in glucose metabolism^14^. Recently, SMEK, the regulatory subunit of PP4, has been shown to participate in hepatic gluconeogenesis via regulating CRTC2 phosphorylation^13^. In our research, we confirmed PP4 contributed to hepatic insulin resistance. PP4 over-expression *in vitro* resulted in reduced glycogen levels and disruption of the insulin signaling, as represented by elevated JNK and IRS-1 (serine 307) phosphorylation, as well as decreased AKT and GSK3β phosphorylation. Yet silencing of PP4 expression caused elevated glycogen levels and ameliorated insulin sensitivity especially in hepatocytes treated with TNF-α. On the other hand, to examine the role of PP4 in hepatic insulin resistance *in vivo*, we established a mouse insulin resistance model by treating C57BL/6J mice with TNF-α. PP4 is a developmentally regulated protein phosphatase and is essential for embryogenesis. Indeed, loss of PP4 led to the embryonic lethality of mice at the early embryonic stage, and any PP4-deficient embryos could not be identified when they were dissected as early as embryonic day 9.5^23^. Here, PP4 expression was suppressed in the livers by AD-PP4 shRNA. The knock-down of PP4 reversed TNF-α-induced impaired insulin signaling. It is noteworthy that pyruvate tolerance and glucose tolerance were ameliorated in the AD-PP4 shRNA-injected mice, indicating ameliorated gluconeogenesis and glucose tolerance in the C57BL/6J mice treated with TNF-α. In addition, insulin tolerance tests showed that PP4 silencing could improve insulin sensitivity. Finally, we explored the effect of PP4 activity on hepatic insulin resistance. Thus far, no specific chemical has been found to inhibit the activity of PP4 specifically. PP4RL, a PP4 phosphatase-dead mutant in which the replacement of arginine 236 with leucine leads to the loss of its phosphatase activity, was widely used to inhibit PP4 activity specifically^10^,^12^. By transfecting PP4RL expression vector into HepG2 cells, PP4 activity was inhibited. The suppression of PP4 activity reversed the reduced glycogen levels and impaired insulin signaling caused by TNF-α. Taken together, these data strongly suggested that PP4 might be a linker between TNF-α and hepatic insulin resistance.

What is the mechanism underlying PP4 involvement in TNF-α-induced hepatic insulin resistance? In the present study, we determined that PP4 is a novel negative regulator of IRS-1 function. Our results demonstrated that: (1) TNF-α triggered IRS-1 phosphorylation on serine 307 and decreased IRS-1 protein level; (2) over-expression of PP4 not only induced IRS-1 phosphorylation and IRS-1 protein reduction, but amplified the effect of TNF-α on IRS-1; (3) suppression of PP4 expression and activity restored the effect of TNF-α on IRS-1. These results showed that PP4 regluated the phosphorylation status and protein level of IRS-1 in TNF-α signaling. The shift of insulin signaling in the layer of IRS proteins is modulated in a very subtle and complex manner using either the phosphorylation pattern or protein levels of IRS as a regulatory mechanism involving tyrosine phosphorylation, serine phosphorylation and ubiquitination^24^,^25^. In our studies, PP4 over-expression-induced IRS-1 hyperphosphorylation on serine 307 and reduced protein level were strongly inhibited by SP600125, suggested that JNK mediated the effect of PP4 on IRS-1. The activation of JNK signaling triggered the serine phosphorylation of IRS-1, which blocked the tyrosine phosphorylation of IRS-1 and reduced the ability of IRS-1 to attract PI3-kinase, thereby blocking insulin signaling. As an important component in insulin signaling cascade, IRS-1 was regulated by a complex mechanism involving phosphorylation of serine/threonine residues^26^. Moreover, it was indicated that the celluar IRS-1 protein level was likely to be regulated by proteasome-mediated degradation^25^,^27^. Proteasome degradation pathway depends mostly on the ubiquitination and some modifications such as serine phosphorylation^28^. Some studies revealed that IRS-1 could be ubiquitinated and subsequently degraded by proteasome during insulin or TNF-α stimulation^29^,^30^,^31^. In addition, through suppression of key phosphorylation sites required for IRS-1 degradation such as S307, the ubiquitination and degradation of IRS-1 was delayed^32^. Combined with our results that hyperphosphorylated IRS-1 on serine 307 site and reduced IRS-1 protein were detected in TNF-α-induced hepatic insulin resistance, we speculated that PP4 promoted IRS-1 phosphorylation through JNK activation, thus resulting in IRS-1 degradation via proteasome degradation system. Moreover, over-expression of PP4 led to increased phosphorylation level of JNK, whereas suppression of PP4 expression and activity restored elevated activation of JNK induced by TNF-α in hepatocytes. These results indicated that PP4 could be a positive regulator of JNK activity. Unfortunately, no direct interaction was observed between PP4 and JNK, raising the possibility that PP4 regulated JNK activation via upstream regulators of JNK. However, the mechanism of JNK activation by PP4 in hepatic insulin resistance is still unknown. There are two possibilities: (1) As a serine/threonine phosphatase, PP4 may dephosphorylate some upstream kinase of JNK, whose activity is inhibited by phosphorylation^10^. (2) PP4 may dephosphorylate some upstream inhibitory kinase to relieve the inhibition of JNK.

**Figure 7:**
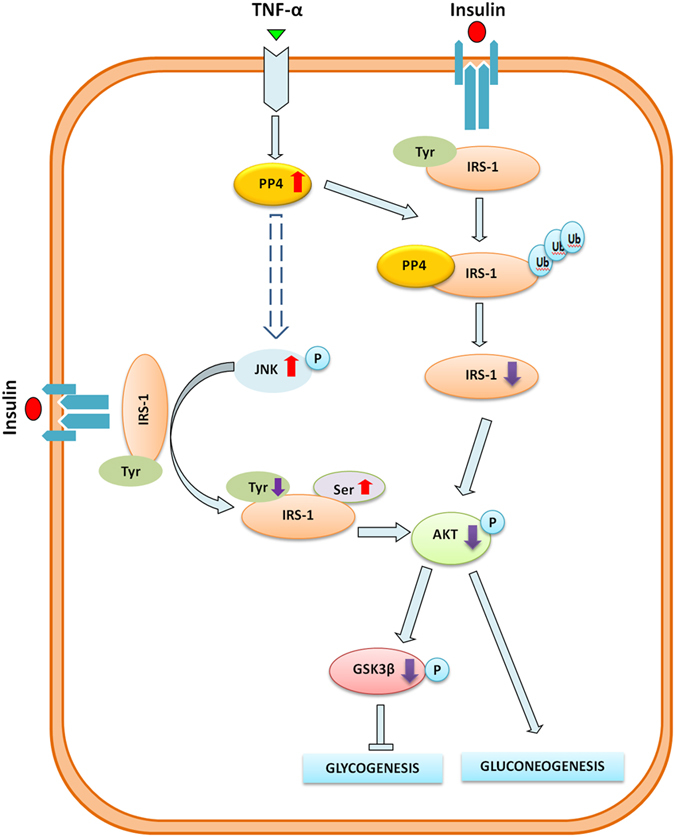
Proposed mechanisms by which PP4 functions as a critical regulator in TNF-α-induced hepatic insulin resistance.

Moreover, we proposed a direct interaction between PP4 and IRS-1. IRS-1 interacted with both exogenous PP4 and endogenous PP4, indicating that PP4 potentially formed a complex with IRS-1. Additionally, we found that PP4 co-localized with IRS-1 in HepG2 cells. Combined with the fact that PP4 can not only activate JNK to induce the phosphorylation of IRS-1 on serine 307, but reduce the expression of IRS-1, we believe that PP4 formed a complex with IRS-1 to promote the phosphorylation of IRS-1 via JNK activation as well as reduce the expression of IRS-1. In conclusion, as shown in Fig. 7, this study provided novel data to demonstrate that PP4 functions as a critical regulator in TNF-α-induced hepatic insulin resistance. Our study suggested that TNF-α impaired the activation of the PI3K/AKT/GSK3β pathway and the synthesis of glycogen through the up-regulation of PP4, accompanied by increased serine 307 phosphorylation of IRS-1 and decreased expression of IRS-1. Moreover, PP4 impaired the insulin signaling by forming a protein complex with IRS-1 to promote IRS-1 phosphorylation on serine 307 via JNK activation as well as down-regulate IRS-1 expression. These findings provide mechanistic insight into the critical role of PP4 in the regulation of the insulin signaling and the synthesis of glycogen in hepatocytes.

## Methods

### Animal Experiment

All animal procedures were performed in accordance with the National Institutes of Health Animal Care and Use Guidelines. All animal protocols were approved by the Animal Ethics Committee at the Beijing Institute of Geriatrics.

db/db mice (n = 10) and age-matched wild-type (WT) mice (n = 10) were fed a standard laboratory diet for 12 weeks, purchased from the Peking University Health Science Center.

Twelve-week-old male C57BL/6J mice matched for weight and glucose level were separated for 6 groups (Control, TNF-α, control adenovirus (AD), TNF-α+ control AD, AD-PP4 shRNA, TNF-α+ AD-PP4 shRNA) with 9 mice per group and fed on a standard laboratory diet.

For chronic TNF-α (California Bioscience, Coachella, CA, USA) exposure, Alzet osmotic pumps (Durect, Cupertino, CA, USA) with a 7-day pumping capacity and infusion rate of 1 μl/h were used. Pumps were filled to capacity with 7.1 μg/ml h TNF-α diluted in carrier (0.9% NaCl and 0.1% BSA)^33^. To knockdown hepatic PP4 expression, the recombinant adenovirus (2 × 10^9^ plaque-forming unit/25 g body weight) was delivered into mice by tail vein injection.

For glucose tolerance tests, pyruvate tolerance tests and insulin tolerance tests, mice were injected i.p. with glucose and pyruvate (2 g/kg of body weight) and insulin (0.5U/kg of body weight) after 8, 16 or 4 h of fasting 5–7 d after injection of adenovirus vectors, respectively. Blood glucose levels were monitored with a glucometer (ACCU-CHEK Advantage, Roche Diagnostics GmbH, Mannheim, Germany) at the indicated time points.

Serum TNF-α and insulin concentrations were determined respectively with an ELISA kit for TNF-α (Uscn Life Science Inc., Wuhan, China) and an insulin ELISA kit (EMD Millipore, Darmstadt, Germany). The insulin sensitivity index was calculated as 1/(fasting insulin concentration × fasting glucose concentration).

### Plasmids, SiRNA and Adenovirus Vectors

Green Fluorescent Protein (GFP)-PP4, PP4-GFP and HA-PP4 were constructed by inserting human PP4 cDNA into the pEGFP-C1, pEGFP-N2 and pHM6 vectors, respectively. DsRed-IRS1 was generated by inserting human IRS-1 cDNA into the pDsRed-N1 vector. FLAG-PP4RL mutants were obtained using the QuickChange site-directed mutagenesis kit (Stratagene, La Jolla, CA, USA) to substitute leucine for the arginine at amino acid 235 in the phosphatase domain of the pEGFP-C1-PP4 construct and subcloned into the p3XFLAG-CMV vector.

Small interfering RNA (siRNA) of PP4 was purchased from Genepharma (Suzhou, Jiangsu, China) and listed in supplementary information Table S1. The pAdxsi-GFP-PP4, pAdxsi-GFP-PP4shRNA and the control adenovirus vector were purchased from the Chinese national human genome center.

### Culture of HepG2 Cells and Mouse Primary Hepatocytes

HepG2 cells were grown in minimum essential medium (GIBCO, Grand Island, NY, USA) supplemented with 10% fetal calf serum at 37 °C in a humidified atmosphere of 5% CO_2_. Mouse primary hepatocytes were isolated as previously described^34^. For TNF-α treatment, HepG2 cells and the hepatocytes were exposed to 10 ng/ml TNF-α for 24 h. For insulin treatment, HepG2 cells and hepatocytes were stimulated to 10 nM insulin for 10 min after serum starvation. For adenovirus vector infection, adenovirus vectors were added for 24 h before TNF-α treatment. For knockdown the PP4 expression, PP4 siRNA purchased from Genepharm. Hiperfect transfection reagent (Qiagen, Hilden, North Rhine-Westphalia, Germany) was used for the transfection of siRNA according to the manufacturer’s instruction.

### Western Blot Analysis

Total protein (20–30 μg) were subjected to 10% SDS-PAGE. Western blot were performed with antibodies for AKT, phosphorylated AKT (Ser473), glycogen synthase kinase3β (GSK3β) and phosphorylated GSK3β (Ser9) (Cell Signaling Technology, Danvers, MA, USA); FLAG (Abcam, Cambridge, MA, USA), HA (Sigma, St.Louis, MO, USA), PP4 (sc-6118) and β-actin (Santa Cruz Biotechnology, Inc., Dallas, TX, USA).

### Quantitative real-time PCR

Total RNA was extracted using TRIzol (Invitrogen, Carlsbad, CA, USA). Quantitative real-time PCR was performed using SYBR Green Realtime PCR Master Mix (Applied Biosystems, Foster City, CA, USA). Primer sequences are shown in supplementary information Table S2.

### Measurement of Glycogen Content

The glycogen levels were measured in the presence of 10nM insulin using a glycogen assay kit (BioVision, San Francisco, CA, USA).

### Immunoprecipitation

The cells were lysed in buffer containing 50 mM Tris-HCl (pH 8.0), 1% Nonidet P-40, 120 mM NaCl, 1 mM EDTA, 6 mM EGTA, 1 mM dithiothreitol, 50 μM PMSF and 2 μg/ml aprotinin. Endogenous PP4 and over-expressed HA-PP4 were immunoprecipitated with an anti-PP4 antibody and anti-HA antibody, respectively.

### Phosphatase Assays

Phosphatase assays were performed using the Ser/Thr phosphatase assay kit 1 (Upstate Biotechnology, Lake Placid, NY, USA), according to the manufacturer’s protocol.

### Statistical Analysis

All values are represented as means ± S.D. of the indicated number of measurements. A one-way analysis of variance test was used to determine significance, requiring *p* < 0.05 for statistical significance.

## Additional Information

**How to cite this article**: Zhao, H. *et al.* Protein phosphatase 4 (PP4) functions as a critical regulator in tumor necrosis factor (TNF)-α-induced hepatic insulin resistance. *Sci. Rep.* **5**, 18093; doi: 10.1038/srep18093 (2015).

## Supplementary Material

Supplementary Information
