# Supplementary material for: Protein phosphatase 4 (PP4) functions as a critical regulator in tumor necrosis factor (TNF)-α-induced hepatic insulin resistance
Source: Sci Rep. 2015 Dec 15;5:18093. doi: 10.1038/srep18093 (PMC4678300; doi:10.1038/srep18093)
Supplement: Supplementary Information [file srep18093-s1.pdf]

Supplementary Information

for

Protein phosphatase 4 (PP4) functions as a critical regulator in tumor necrosis factor (TNF)-α-induced hepatic insulin resistance

Hongye Zhao[1,2,4], Xiuqing Huang[2,4] , Juan Jiao[1,2] , Hangxiang Zhang[2] , Jin Liu[3] , Weiwei Qin[2] , Xiangyu Meng[2] , Tao Shen[2] , Yajun Lin[2] , Jiaojiao Chu[2] & Jian Li[1,2,*]

[1] Graduate School of Peking Union Medical College and Chinese Academy of Medical Sciences, Beijing 100730, China

[2] The Key Laboratory of Geriatrics，Beijing Hospital & Beijing Institute of Geriatrics, Ministry of Health, Beijing 100730, China

[3] College of Life Sciences, Beijing Normal University, 100875, P.R. China

[4] Co-first authors

*Correspondence: lijian@bjhmoh.cn

Supplementary Fig. S1

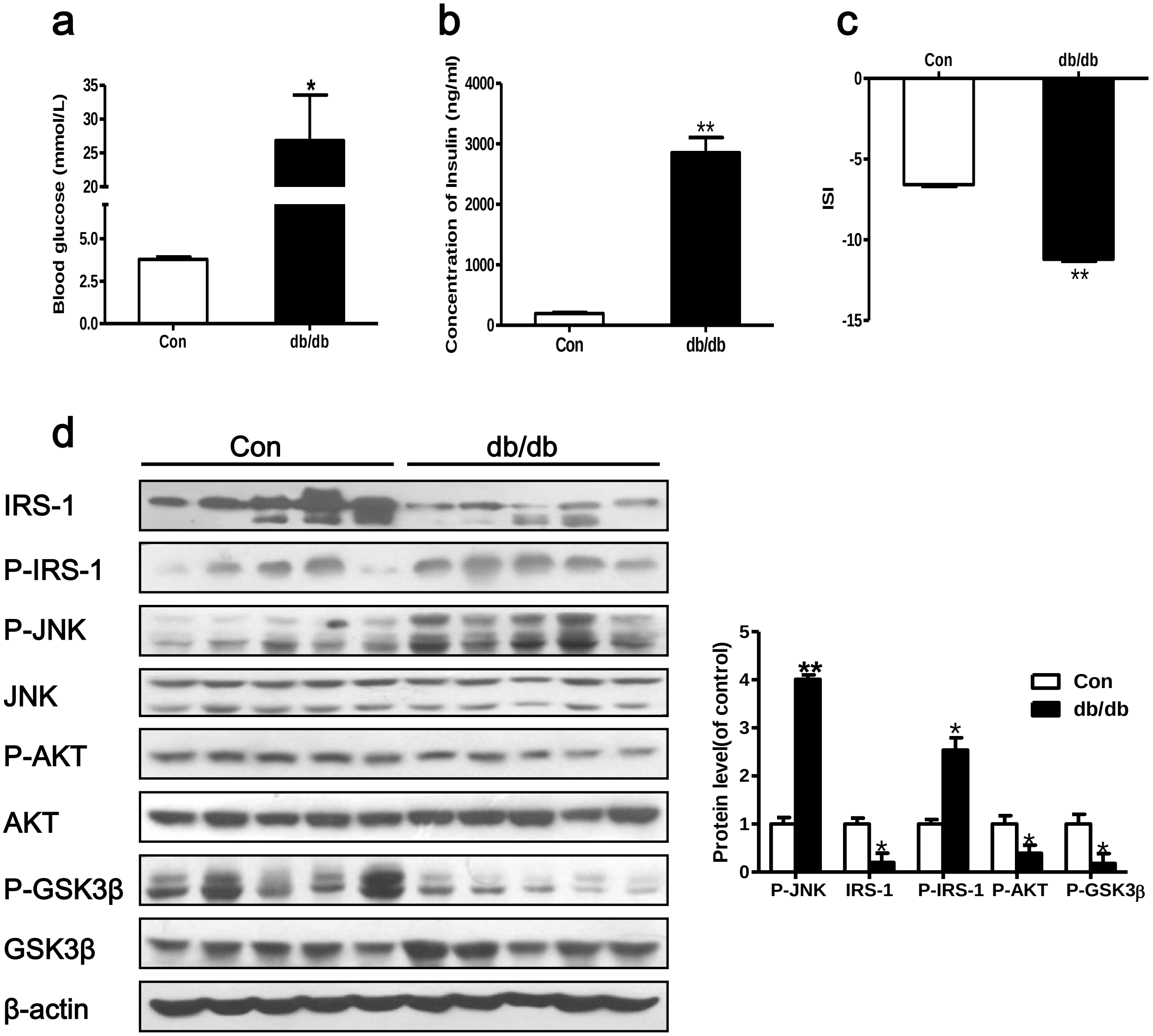

Figure S1. db/db mice exhibited obvious features of insulin resistance. The levels of blood glucose (**a**) and insulin concentration (**b**) were increased, whereas the insulin sensitive index (ISI) was decreased (**c**). The insulin signaling pathway was disordered in the livers of db/db mice, assessed by increased phosphorylation of IRS-1 (ser 307) and JNK, reduced expression of IRS-1 and AKT/GSK3β phosphorylation (**d**). Data represent the means ± S.D., (n=10 mice *in vivo*). *P<0.05 and **P<0.01 versus control. Con ─ control.

Supplementary Fig. S2

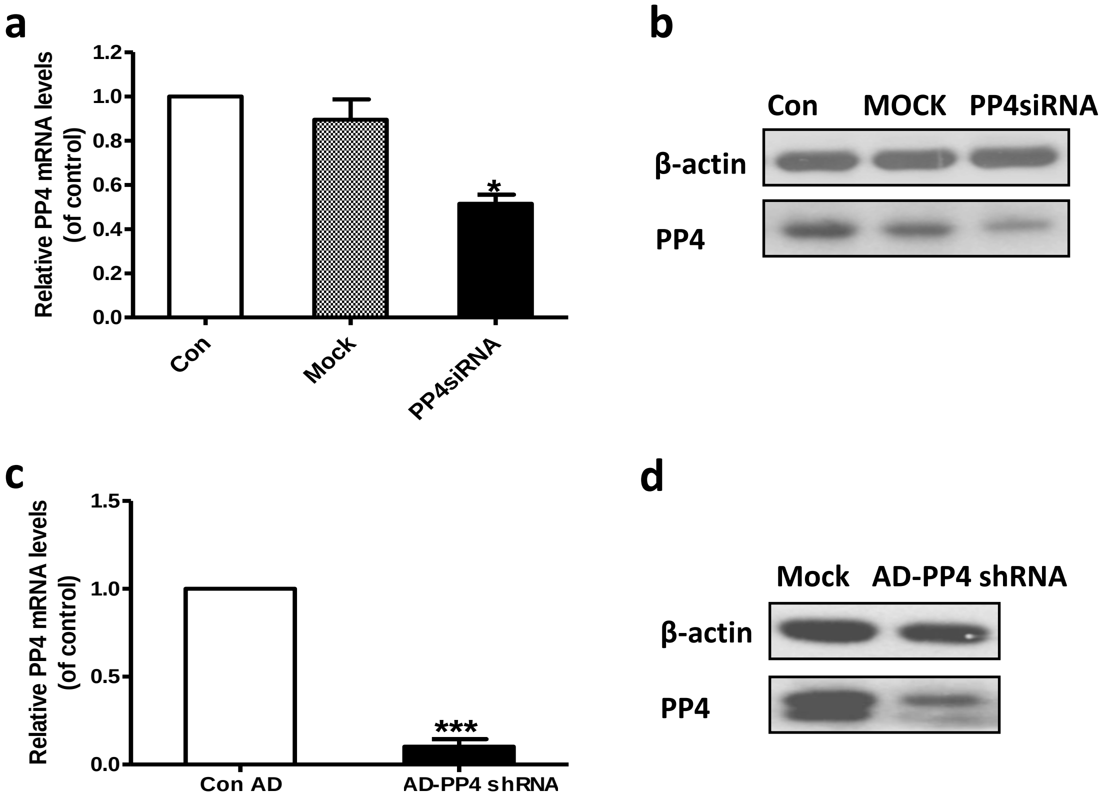

Figure S2. Expression of PP4 was decreased by PP4 siRNA and AD-PP4 shRNA. In order to knock down the expression of PP4, HepG2 cells and mouse primary hepatocytes were transfected with PP4siRNA or AD-PP4 shRNA, respectively. By applying quantitative real-time PCR and western blot analysis, we detected both PP4 siRNA (**a, b**) and AD-PP4 shRNA (**c, d**) could reduce the expression of PP4. Data represent the means ± S.D., (n=3 independent experiments in vitro). *P<0.05 and ***P<0.001 versus control. Con ─ control; Con AD ─ control AD.

Supplementary Table S1. Oligonucleotides of PP4siRNA

Gene Name	Sequences (5’ 〉 3’)
PP4C-728s	GGUUACAAGUGGCACUUCATT
PP4C-728as	UGAAGUGCCACUUGUAACCTT

Supplementary Table S2. List of oligonucleotide primers for quantitative real-time PCR

Gene Name	Forward primer (F)<br>Reverse primer (R)	Primer' Sequences (5' 〉 3')
18s rRNA	*F*	GTAACCCGTTGAACCCCATT
18s rRNA	*R*	CCATCCAATCGGTAGTAGCG
Human PP4	*F*	CTGACCCAGAAGACACCACA
Human PP4	*R*	TGAGCACCGTCTCATTGAAG
Human G6Pase	*F*	TACGTCCTCTTCCCCATCTG
Human G6Pase	*R*	CCTGGTCCAGTCTCACAGGT
Human PEPCK	*F*	GAGAGAACTCCAGGGTGCTG
Human PEPCK	*R*	CCTTGGAGATGCTGAAAAGC
Human PGC-1α	*F*	CACCAGCCAACACTCAGCTA
Human PGC-1α	*R*	ACGTCTTTGTGGCTTTTGCT
Mouse PP4	*F*	CAATCATGAGAGTCGCCAGA
Mouse PP4	*R*	GATGGAAGGGGAAAGACCTC
Mouse G6pase	*F*	TCTGTCCCGGATCTACCTTG
Mouse G6pase	*R*	GTAGAATCCAAGCGCGAAAC
Mouse Pepck	*F*	TCTGAGATCTCTGATCCAGACC
Mouse Pepck	*R*	GAAGTCCAGACCGTTATGCAGC
Mouse Pgc-1α	*F*	GCCTATGAGCACGAAAGGC
Mouse Pgc-1α	*R*	TCACACGGCGCTCTTCAATT
Mouse Tnf-α	*F*	CACAGAAAGCATGATCCGCG
Mouse Tnf-α	*R*	ACTGATGAGAGGGAGGCCAT
